# The Development of Nomograms to Predict Blastulation Rate Following Cycles of *In Vitro* Fertilization in Patients With Tubal Factor Infertility, Polycystic Ovary Syndrome, or Endometriosis

**DOI:** 10.3389/fendo.2021.751373

**Published:** 2021-11-03

**Authors:** Haixia Jin, Xiaoxue Shen, Wenyan Song, Yan Liu, Lin Qi, Fuli Zhang

**Affiliations:** Reproductive Medicine Center, The First Affiliated Hospital of Zhengzhou University, Zhengzhou, China

**Keywords:** *in vitro* fertilization, blastocyst, tubal factor infertility, endometriosis, polycystic ovary syndrome (PCOS), predictive models

## Abstract

It is well known that the transfer of embryos at the blastocyst stage is superior to the transfer of embryos at the cleavage stage in many respects. However, the rate of blastocyst formation remains low in clinical practice. To reduce the possibility of wasting embryos and to accurately predict the possibility of blastocyst formation, we constructed a nomogram based on range of clinical characteristics to predict blastocyst formation rates in patients with different types of infertility. We divided patients into three groups based on female etiology: a tubal factor group, a polycystic ovary syndrome group, and an endometriosis group. Multiple logistic regression was used to analyze the relationship between patient characteristics and blastocyst formation. Each group of patients was divided into a training set and a validation set. The training set was used to construct the nomogram, while the validation set was used to test the performance of the model by using discrimination and calibration. The area under the curve (AUC) for the three groups indicated that the models performed fairly and that calibration was acceptable in each model.

## Introduction

Assisted reproductive technology (ART) has become an important tool to help infertile couples successfully conceive children. Globally, it is estimated that there have been at least eight million babies born through ART since the very first *in vitro* fertilization–embryo transfer (IVF-ET) procedure in 1978 (1). Although ART has developed rapidly over the past 43 years, clinical pregnancy rates and live birth rates are still low. Numerous studies have found that the transfer of embryos at the blastocyst stage is superior to the transfer of embryos at the cleavage stage in many different aspects. For example, transfer at the blastocyst stage allows for the natural screening of embryos; this means that high-quality embryos can be selected following activation of the embryonic genome. A previous study involving women over the age of 36 years with three or more failed IVF treatments showed that almost half of the morphologically normal embryos on day 3 had chromosomal abnormalities (2). However, Staessen et al. observed an increase in the probability of selecting chromosomally normal embryos from 41.3% at the cleavage stage to 65% at the blastocyst stage (3). The transfer of embryos at the blastocyst stage enables embryo development to synchronize better with the endometrial implantation window and is more in line with the physiological implantation time. When fertilized *in vivo*, embryos remain within the fallopian tube on day 3; however, when an embryo is transferred on day 3 of development, it is placed directly into the uterine cavity and not the fallopian tube. Since the uterine cavity provides a different nutritional environment than the fallopian tubes, it is likely that the process of embryo transfer will affect embryo implantation (4). It is too early to expose embryos on day 3 to the uterine environment, especially those that suffer from excessive stimulation and high levels of estrogen (5). In a previous study, Glujovsky et al. reported that the transfer of embryos at the blastocyst stage resulted in higher live birth rates than the transfer of embryos at the cleavage stage (6). In addition, an increasing number of reproductive medicine centers around the world now prefer to choose single embryo transfer because multiple pregnancies are associated with many complications. Single blastocyst transfer reduces the risk of multiple births but does not reduce the pregnancy outcome (7).

However, the successful development of embryos from the cleavage stage to the blastocyst stage is a risky process, and failure to develop into blastocysts can result in the cancellation of embryo transfers (8) and financial loss and creates psychological stress for the patients. In an attempt to enhance the rate of blastocyst formation, a range of techniques are currently being used to select embryos at the cleavage stage that exhibit higher development potential for further culture to the blastocyst stage, including embryo morphology, time-lapse imaging, the analysis of metabolites, and preimplantation genetic testing (PGT). However, these methods rely on subjective evaluation by embryologists and can increase the financial burden for patients. Although an increasing number of couples are giving birth by transferring embryos at the blastocyst stage each year, many embryos at the cleavage stage are still wasted due to prolonged culture failure. Consequently, there are still outstanding issues with regard to reducing the cancellation rate of blastocyst transfer.

A nomogram based on logistic regression analysis and multiple factors can provide accurate predictions for various situations (9). The ability of such nomograms to predict the diagnosis, staging, and prognosis of a disease has been confirmed to be better than that of other predictive models, such as risk stratification and artificial neural networks (10). Furthermore, a nomogram can convert influential factors into a number of components that can intuitively predict outcomes; the total score yielded by such nomograms represent the probability of an outcome.

Nomogram has been applied to the field of ART in recent years. For example, Wu et al. constructed a nomogram to predict the clinical pregnancy of women undergoing fresh embryo transfer treatment (11). In addition, a previous study designed a nomogram to predict the cancellation of blastocyst transfer but did not consider the etiology of female infertility or specific outcomes (12). In the present study, we adapted the previously developed predictive model by including the effects of a range of female factors on blastocyst formation. This new model provides accurate reference guidelines for patients and doctors and will facilitate the management of IVF-ET cycles.

## Materials and Methods

### Patients

**Figure 1:**
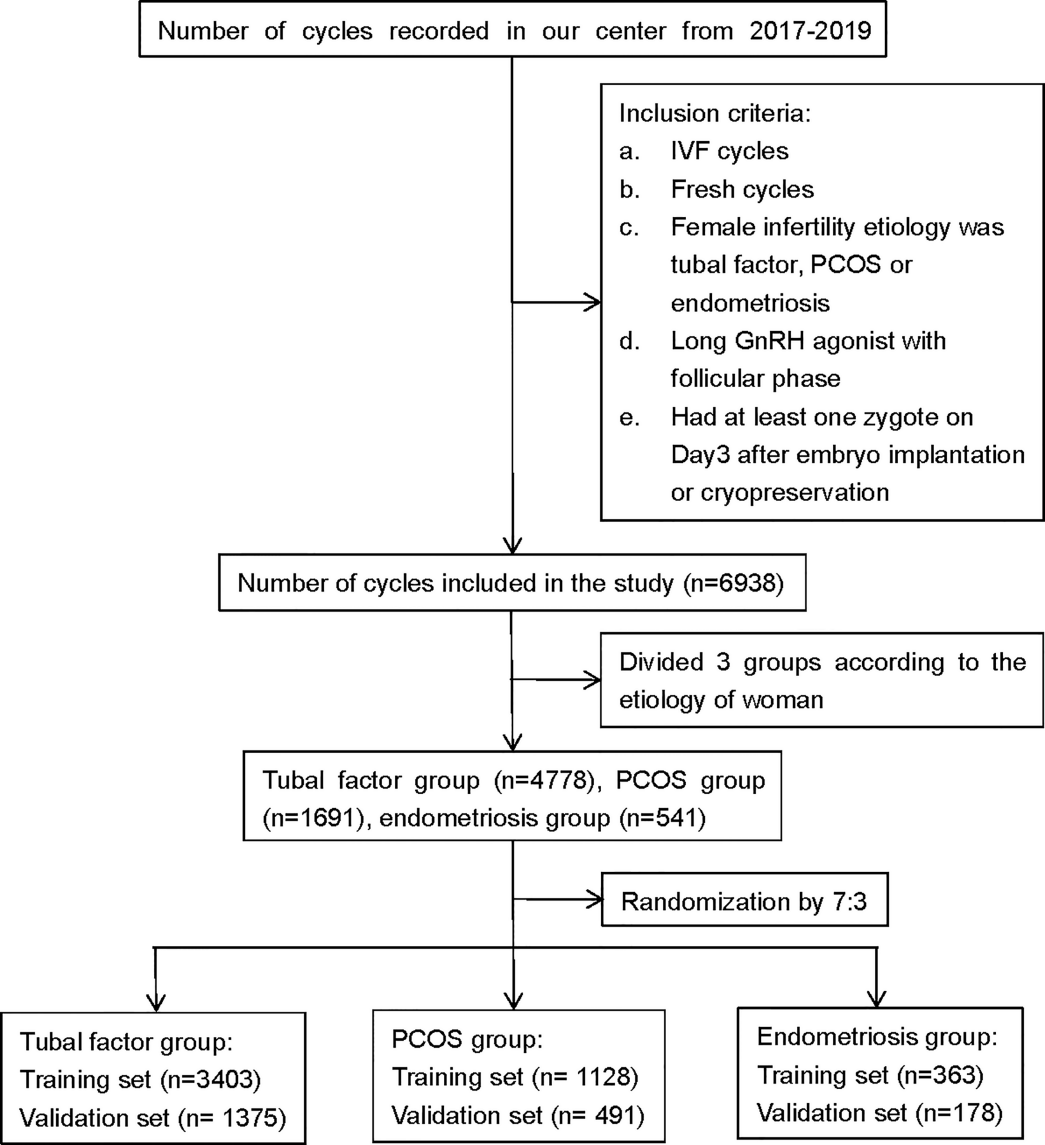
Flowchart showing data selection process. PCOS, polycystic ovary syndrome; GnRH, gonadotropin-releasing hormone; IVF, *in vitro* fertilization.

We retrospectively analyzed the clinical and laboratory data of patients who had undergone IVF treatment at the Reproductive Medicine Center of the First Affiliated Hospital of Zhengzhou University, Henan, China, between January 2017 and December 2019. All patients were treated with the long follicular phase gonadotropin (Gn)-releasing hormone (GnRH) agonist protocol for three main types of infertility: tubal factors, polycystic ovary syndrome (PCOS), or endometriosis. Patients were included if they had IVF treatment cycles, fresh cycles, or at least one zygote on day 3 after embryo implantation, or had embryos cryopreserved. Patients were excluded if they had received cycles with surgically retrieved sperm, oocyte donation, intracytoplasmic sperm injection (ICSI), and PGT. The patients included in the analysis were divided into three groups according to the type of infertility: a tubal factor group, a PCOS group, and an endometriosis group. **Figure 1** shows a flowchart depicting the selection process and the inclusion criteria. Tubal infertility was diagnosed by imagining examinations; PCOS was diagnosed by the Rotterdam criteria (13); and endometriosis was diagnosed by physical examination, transvaginal sonography, and MRI using previously published criteria (14). All patients were informed that their personal medical history would be analyzed and the study was approved by the Ethics Committee of the First Affiliated Hospital of Zhengzhou University (Reference: 2021-KY-0023-001).

### *In Vitro* Fertilization Procedures

The ovarian stimulation protocol used in our study involved a long follicular phase GnRH agonist. A long GnRH agonist (3.75 mg, IPSEN, France) was given on days 2 and 3 of menstruation. After 30–42 days, patients reached the downregulation standard (no follicles >10 mm in diameter; estradiol <183 pmol/L, luteinizing hormone (LH) <3 IU/L). The dosage of Gn was then adjusted according to body mass index (BMI), ovarian reserve, and ovarian responses to perform controlled ovarian stimulation (COS). Human chorionic Gn (hCG) (2000IU, LIVZON, China) and recombinant human choriogonadotropin (250 μg, Merck, Italy) were administered when the following criteria were met: 1 dominant follicle ≥20 mm in diameter, 3 follicles ≥17 mm in diameter, or 2/3 follicles r ≥16 mm in diameter. Following the injection of hCG at 37 h, the follicles were punctured under the guidance of transvaginal ultrasound, and oocyte corona cumulus complexes (OCCCs) were visualized with a stereoscopic microscope. The OCCCs were rapidly transferred to G-MOPS Plus (Vitrolife, Sweden) and washed three times in G-IVF Plus (Vitrolife, Sweden). Finally, the OCCCs were transferred into G-IVF plus culture medium in an incubator at 37°C with 6% CO_2_ and 5% O_2_ to await insemination (day 1). The day of oocyte retrieval was designated as day 0.

Semen samples underwent density gradient centrifugation, and sperm were then added to 50 μl of G-IVF Plus microdroplet medium following hCG injection at 39–40 h. In total, 10,000 sperm were added to each oocyte. Sperm and oocytes were co-cultured for 5 h; oocytes and cumulus cells were then mechanically dissected with a 150-μm needle and then observed for formation of the second polar body. If >30% of the fertilized oocytes were shown to form a second polar body, then the zygotes were transferred to medium containing G-1 plus (Vitrolife, Sweden) until day 3. In addition to embryo transfer and freezing on day 3, the remaining embryos showing signs of normal fertilization were transferred to a medium containing G-2 Plus (Vitrolife, Sweden) until day 5/6 in an incubator at 37°C with 6% CO_2_ and 5% O_2_.

In our center, we grade embryos on day 3 according to the Peter scoring system (15); the Gardner scoring system (16) was used to score blastocysts. In this study, all embryos with a score of I or II were considered as top-quality embryos; all embryos consisting of 6–10 cells and defined as having a Gardner score ≥3BB were considered as high-quality blastocysts.

### Statistical Analysis

The measurable outcome of this study was the probability of extended culture to the blastocyst stage following an IVF cycle. Differences in blastocyst formation rates between the three groups were calculated to determine whether the division of patients into three groups based on the type of infertility was statistically significant. Levene’s test was used to test the homogeneity of variance. Due to clear heterogeneity of variance, the Kruskal–Wallis test was used to compare the blastocyst formation rate between the three groups. Finally, Dunnett’s t-test was used to determine whether there was any statistical difference in blastocyst formation rate between any two groups.

Multivariable logistic regression (MLR) analysis was used to test the association between blastulation rate and patient characteristics. Backwards stepwise variable selection was performed to determine independent variables. We included a range of variables in the models, including women’s age and BMI, serum anti-Mullerian hormone (AMH) levels, basal levels of follicle-stimulating hormone (FSH), the duration of infertility, the dose of Gn, the number of oocytes retrieved, the rate of top-quality embryos cultured on day 3, and the fertilization rate. Variables were eliminated from the model if their removal led to an improvement in the overall quality of the model (as measured by the Akaike information criterion). p-Values were based on Wald’s tests in the multivariable analysis. p-Values <0.05 were significant.

Nomograms were constructed by using the MLR to delineate and visualize the models generated. The discrimination and calibration abilities of each model were assessed by calculating the area under the curve (AUC) of the receiver operating characteristic (ROC) and calibration curves (17). The AUC is a summary measure of the ROC and reflects the ability of a test to discriminate the outcomes across all possible levels of positivity. A 95% CI was calculated for the AUC. AUCs range from 0 to 1; and a model is considered to have poor, fair, or good performance if the AUC lies between 0.5 and 0.7, 0.7 and 0.8, or 0.8 and 0.9, respectively (18). Next, we plotted several quantiles of the empirical distribution for predictive probability (i.e., predicted probabilities) to construct calibration curves, and we recorded the frequencies observed in each quantile. A perfectly accurate prediction model would result in a plot where the observed and predicted probabilities fell along the diagonal. All analyses were conducted by R software (version 4.0.4; http://www.r-project.org/).

## Results

### Patient Characteristics

We screened all relevant patients attending at our center between 2017 and 2019 and recruited 4,778 patients in the tubal factor group, 1,619 patients in the PCOS group, and 541 patients in the endometriosis group. Patients in each group were randomly sampled into a 7:3 ratio, with 70% as the training set and 30% as the validation set. The training cohort in the tubal factor group, PCOS group, and endometriosis group included 3,403 cycles, 1,128 cycles, and 363 cycles, respectively; the validation cohort included 1,375 cycles, 491 cycles, and 178 cycles, respectively. The training set was used to build the model, and the validation set was used to test the model. The patient and cycle characteristics of the validation set and training set for the three groups are summarized in **Tables 1**–**3**. There were no statistically significant differences in terms of the patient characteristics when compared between the two cohorts within each group.

**Table 1:** Characteristics of tubal factor group in the training and validation cohorts.

	Training cohort	Validation cohort	p-Value
Patients	3,403	1,375	
Female age (years)	31.07 ± 4.73	30.83 ± 4.69	0.116
Male age (years)	31.96 ± 5.50	31.67 ± 5.38	0.103
Female BMI (kg/m^2^)	22.83 ± 3.05	22.85 ± 3.06	0.889
Infertility duration (months)	40.59 ± 36.36	41.97 ± 37.93	0.243
AMH (ng/ml)	3.50 ± 2.45	3.39 ± 2.22	0.123
Basal FSH (mIU/ml)	6.74 ± 1.93	6.69 ± 1.85	0.485
Basal LH (mIU/ml)	5.41 ± 3.64	5.33 ± 3.51	0.477
Gonadotrophin duration (days)	13.48 ± 1.93	13.49 ± 1.93	0.843
Gonadotrophin dosage (IU)	2,492.70 ± 972.80	2,517.50 ± 971.42	0.425
Oocytes retrieved	14.95 ± 6.41	14.86 ± 6.52	0.655
Fertilization rate (%)	65.31 ± 16.55	64.68 ± 17.11	0.242
Cleavage rate (%)	98.82 ± 4.11	98.70 ± 4.54	0.378
Top day 3 embryo rate (%)	64.41 ± 25.51	63.64 ± 26.28	0.349
Blastulation rate (%)	47.93 ± 34.45	47.57 ± 35.15	0.741
High-quality blastulation rate (%)	14.41 ± 22.69	14.80 ± 23.87	0.591
Embryo utilization rate (%)	56.76	57.10	0.514
Average number of ET	1.69 ± 0.46	1.72 ± 0.45	0.069
Embryo implantation rate (%)	47.62	48.10	0.726
Clinical pregnancy rate (%)	62.98	64.36	0.428
Live birth rate (%)	51.17	54.02	0.114

BMI, body mass index; AMH, anti-Mullerian hormone; FSH, follicle-stimulating hormone; LH, luteinizing hormone; ET, embryo transfer.

**Table 2:** Characteristics of PCOS group in the training and validation cohorts.

	Training cohort	Validation cohort	p-Value
Patients	1,128	491	
Female age (years)	28.90 ± 3.72	28.95 ± 3.83	0.809
Male age (years)	29.78 ± 4.24	29.98 ± 4.35	0.397
Female BMI (kg/m^2^)	24.41 ± 3.37	24.40 ± 3.28	0.962
Infertility duration (months)	47.51 ± 33.58	45.66 ± 33.26	0.307
AMH (ng/ml)	8.46 ± 4.32	8.60 ± 4.35	0.548
Basal FSH (mIU/ml)	5.80 ± 1.58	5.80 ± 1.49	0.947
Basal LH (mIU/ml)	10.18 ± 7.82	10.49 ± 7.44	0.463
Gonadotrophin duration (days)	14.65 ± 2.61	14.83 ± 2.77	0.204
Gonadotrophin dosage (IU)	2,174.58 ± 856.0	2,214.03 ± 915.26	0.404
Oocytes retrieved	19.17 ± 7.67	19.24 ± 7.77	0.857
Fertilization rate (%)	64.12 ± 16.71	62.97 ± 16.53	0.200
Cleavage rate (%)	98.70 ± 4.21	98.75 ± 3.63	0.810
Top day 3 embryo rate (%)	66.09 ± 24.70	64.43 ± 24.59	0.215
Blastulation rate (%)	53.30 ± 31.28	52.78 ± 30.20	0.753
High-quality blastulation rate (%)	18.53 ± 23.42	17.93 ± 21.87	0.628
Embryo utilization rate (%)	53.89	53.50	0.618
Average number of ET	1.57 ± 0.50	1.57 ± 0.50	0.979
Embryo implantation rate (%)	61.34	57.76	0.197
Clinical pregnancy rate (%)	76.79	73.84	0.331
Live birth rate (%)	58.39	58.42	0.993

PCOS, polycystic ovary syndrome; BMI, body mass index; AMH, anti-Mullerian hormone; FSH, follicle-stimulating hormone; LH, luteinizing hormone; ET, embryo transfer.

**Table 3:** Characteristics of endometriosis group in the training and validation cohorts.

	Training cohort	Validation cohort	p-Value
Patients	363	178	
Female age (years)	30.78 ± 4.05	30.96 ± 4.43	0.631
Male age (years)	31.82 ± 4.70	31.86 ± 4.87	0.934
Female BMI (kg/m^2^)	22.04 ± 3.17	22.18 ± 3.16	0.612
Infertility duration (months)	41.72 ± 32.18	38.71 ± 29.63	0.295
AMH (ng/ml)	3.02 ± 2.40	3.02 ± 2.09	0.983
Basal FSH (mIU/ml)	6.97 ± 2.10	7.11 ± 2.69	0.500
Basal LH (mIU/ml)	5.54 ± 5.13	5.93 ± 6.11	0.437
Gonadotrophin duration (days)	13.12 ± 1.95	13.21 ± 1.98	0.618
Gonadotrophin dosage (IU)	2,604.65 ± 1,036.67	2,572.75 ± 1,016.47	0.735
Oocytes retrieved	13.58 ± 6.60	14.42 ± 6.83	0.171
Fertilization rate (%)	66.10 ± 18.11	66.77 ± 18.18	0.684
Cleavage rate (%)	99.12 ± 3.14	98.49 ± 4.58	0.097
Top day 3 embryo rate (%)	61.32 ± 27.55	59.37 ± 27.67	0.441
Blastulation rate (%)	44.06 ± 35.50	43.45 ± 35.70	0.849
High-quality blastulation rate (%)	12.10 ± 20.70	12.15 ± 21.20	0.979
Embryo utilization rate (%)	56.50	56.48	0.990
Average number of ET	1.74 ± 0.44	1.72 ± 0.45	0.581
Embryo implantation rate (%)	47.78	42.91	0.210
Clinical pregnancy rate (%)	62.81	54.86	0.112
Live birth rate (%)	46.31	41.67	0.360

Numbers are means ± SD. Basal FSH and LH were measured between the second and third days of female menstrual cycle. No significant difference was found between the training and validation cohorts in the three groups. When p-value is 0.05, we consider it to be significant.

ET, embryo transfer; BMI, body mass index; AMH, anti-Mullerian hormone; FSH, follicle-stimulating hormone; LH, luteinizing hormone.

### Blastocyst Formation Rate in the Three Groups

**Figure 2:**
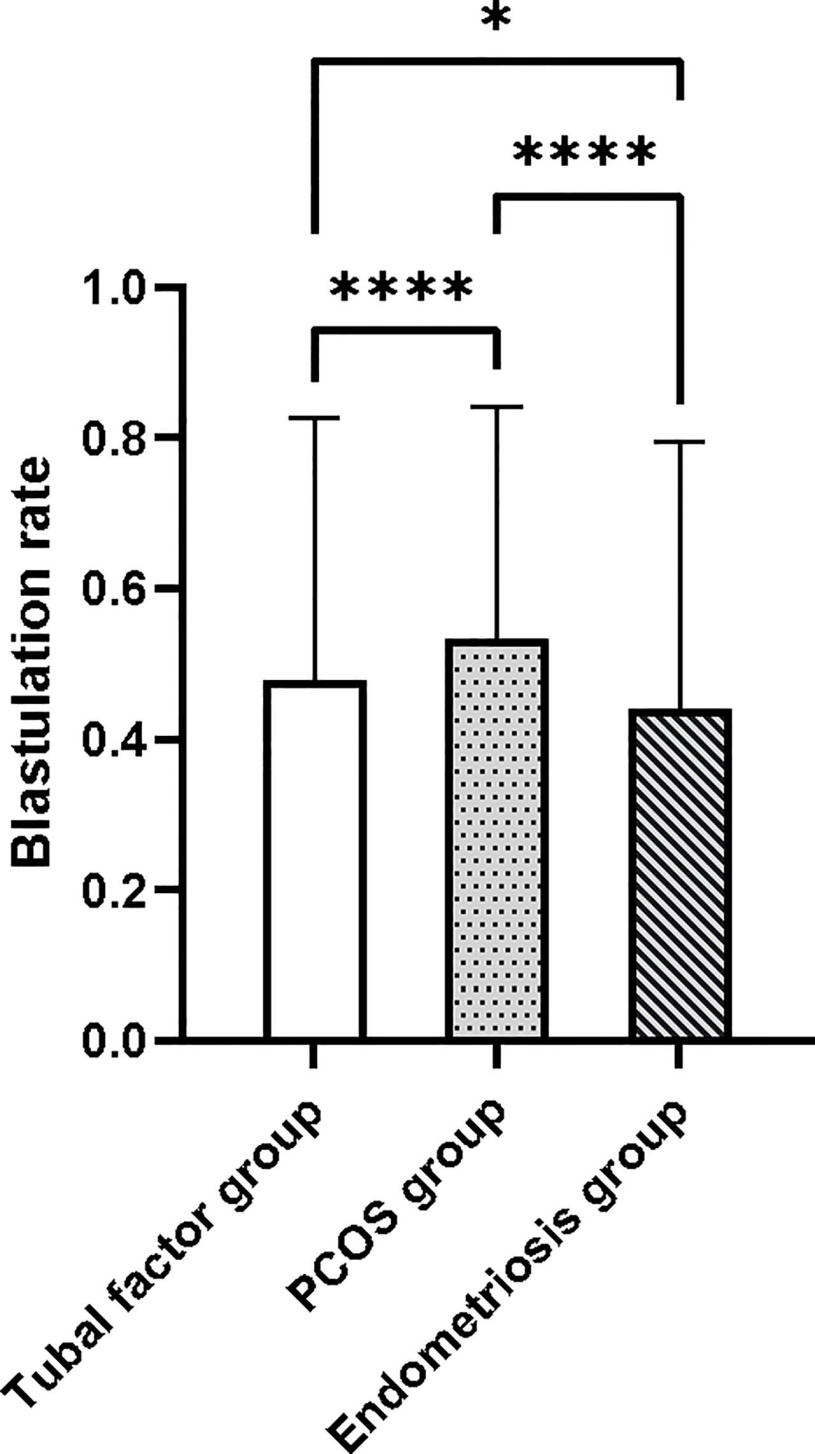
The blastocyst formation rate was significantly different between the tubal factor group, the PCOS group, and endometriosis group by Dunnett’s t-test pair comparison. *p < 0.05, ****p < 0.001. PCOS, polycystic ovary syndrome.

First, we carried out Levene’s test (F = 20.989, p < 0.001) and the Kruskal–Wallis test (H = 39.494, p < 0.001). These results indicated a significant difference in blastocyst formation rate between the three groups. The blastocyst formation rate was significantly different between the tubal factor group and the PCOS group, as determined by Dunnett’s t-test pair comparison (p < 0.001); the rate of blastocyst formation also differed significantly between the tubal factor group and the endometriosis group (p = 0.039). There was also a significant difference in blastocyst formation rate between the PCOS group and the endometriosis group (p < 0.001). Blastocyst formation rates for the three groups are shown in **Figure 2**. The highest blastocyst formation rate was 53% in the PCOS group, followed by 48% in the tubal factor group and 44% in the endometriosis group. There were statistically significant differences in blastocyst formation rate when compared between the three groups.

### Risk Factors for Blastocyst Formation

MLR analysis showed that the blastocyst formation rate was related to female age (odds ratio [OR] = 0.971; 95% CI: 0.955–0.987; p < 0.001), serum AMH (OR = 1.040; 95% CI: 1.001–1.075; p < 0.05), the number of oocytes retrieved (OR = 1.055; 95% CI: 1.041–1.069; p < 0.001), fertilization rate (OR = 7.243; 95% CI: 4.495–11.724; p < 0.001), and the rate of top-quality embryos on day 3 (OR = 14.698; 95% CI: 10.511–20.694; p < 0.001) in the tubal factor group. In the PCOS group, the variables that filtered into the model were the number of oocytes retrieved (OR = 1.048; 95% CI: 1.029–1.068; p < 0.001), fertilization rate (OR = 18.445; 95% CI: 7.913–43.848; p < 0.001), and the rate of top-quality embryos on day 3 (OR = 44.769; 95% CI: 24.239–84.872; p < 0.001). Although female age (OR = 0.982; 95% CI: 0.948–1.017; p = 0.313) and serum levels of AMH (OR = 1.023; 95% CI: 0.992–1.055; p = 0.157) were not statistically different with regard to outcome, they were clinically relevant, and their inclusion did improve the overall quality of the model.

In the endometriosis group, MLR analysis showed that the number of oocytes retrieved (OR = 1.054; 95% CI: 1.009–1.101; p < 0.05), fertilization rate (OR = 27.205; 95% CI: 6.348–125.487; p < 0.001), and the rate of top-quality embryos on day 3 (OR = 8.289; 95% CI: 3.219–22.455; p < 0.001) were significantly related with outcome. Although female age (OR = 0.977; 95% CI: 0.922–1.034; p = 0.414) and serum AMH levels (OR = 1.021; 95% CI: 0.916–1.145; p = 0.711) were not statistically significant, they were included in the model because they were clinically associated with blastocyst formation. **Table 4** shows the variability in ORs and 95% CIs.

**Table 4:** Multivariate regression analysis in predictive factors of blastocyst formation in the training cohorts.

Variables	Tubal factor group		PCOS group		Endometriosis group	
	OR (95% CI)	p-Value	OR (95% CI)	p-Value	OR (95% CI)	p-Value
Female age	0.971 (0.955–0.987)	<0.001	0.982 (0.948–1.017)	0.313	0.977 (0.922–1.034)	0.414
AMH	1.040 (1.007–1.075)	<0.05	1.023 (0.992–1.055)	0.157	1.021 (0.916–1.145)	0.711
Oocytes retrieved	1.055 (1.041–1.069)	<0.001	1.048 (1.029–1.068)	<0.001	1.054 (1.009–1.101)	<0.05
Fertilization rate	7.243 (4.495–11.724)	<0.001	18.445 (7.913–43.848)	<0.001	27.205 (6.348–125.487)	<0.001
Top Day 3 embryo rate	14.698 (10.511–20.694)	<0.001	44.769 (24.239–84.872)	<0.001	8.289 (3.219–22.455)	<0.001

OR, odds ratio; PCOS, polycystic ovary syndrome; AMH, anti-Mullerian hormone.

### Evaluation Forecast Model

**Figure 3:**
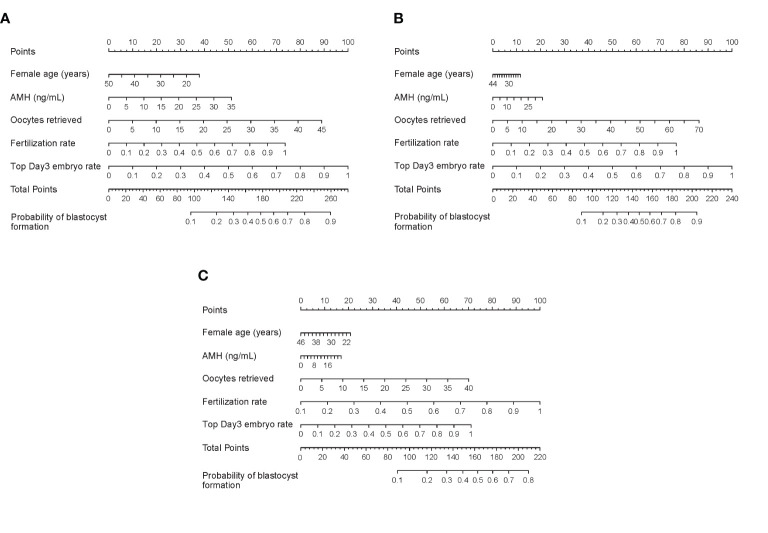
Nomograms to predict blastulation rate after IVF cycle in patients with tubal factor infertility **(A)**, PCOS **(B)**, or endometriosis **(C)**. To read the nomograms, the predicted score for each variable is based on the upper points ruler. The total points were calculated by adding the point of each variable, and the individual probability of blastocyst formation is obtained by a vertical line projected from the total points line to the bottom line. PCOS, polycystic ovary syndrome; AMH, anti-Mullerian hormone; IVF, *in vitro* fertilization.

**Figure 4:**
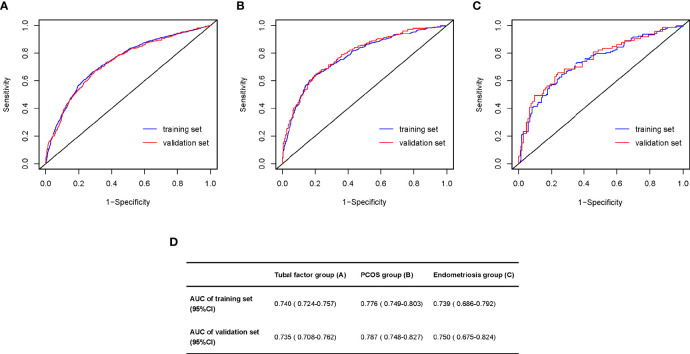
Area under the ROC curve (AUC) for prediction the blastulation rate of infertility women with tubal factor **(A)**, PCOS **(B)**, or endometriosis **(C)**. **(D)** The table shows the value of AUC both training set and validation set in the tubal factor group **(A)**, PCOS group **(B)**, or endometriosis group **(C)**. ROC, receiver operator curve; PCOS, polycystic ovary syndrome.

**Figure 5:**
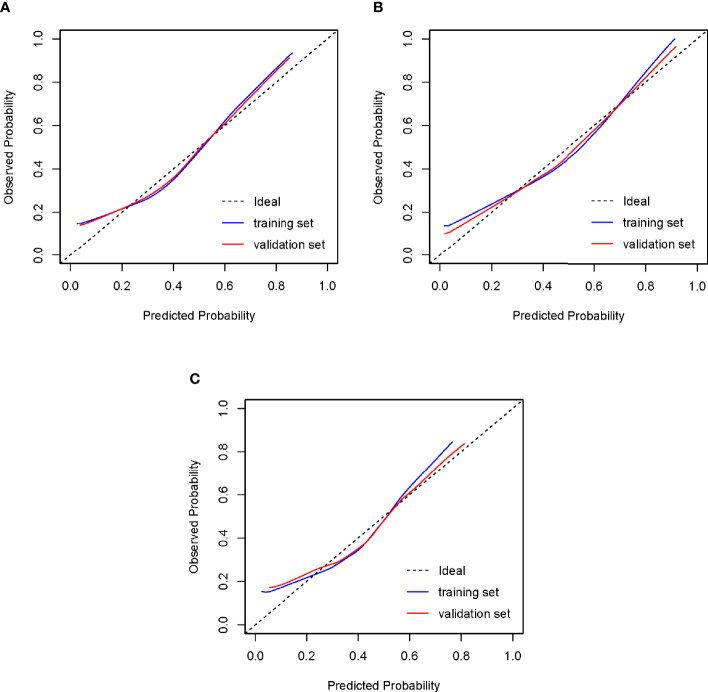
Calibration of the models to predict blastocyst formation *in vitro*. The diagonal lines in the figures represent ideal prediction models. The blue lines and the red lines represent the actual prediction models of the training sets and the validation sets, respectively. The closer their proximity to the diagonal lines, the higher the prediction efficiency of the models. **(A)** Tubal factor group; **(B)** PCOS group; **(C)** endometriosis group. PCOS, polycystic ovary syndrome.

**Figure 6:**
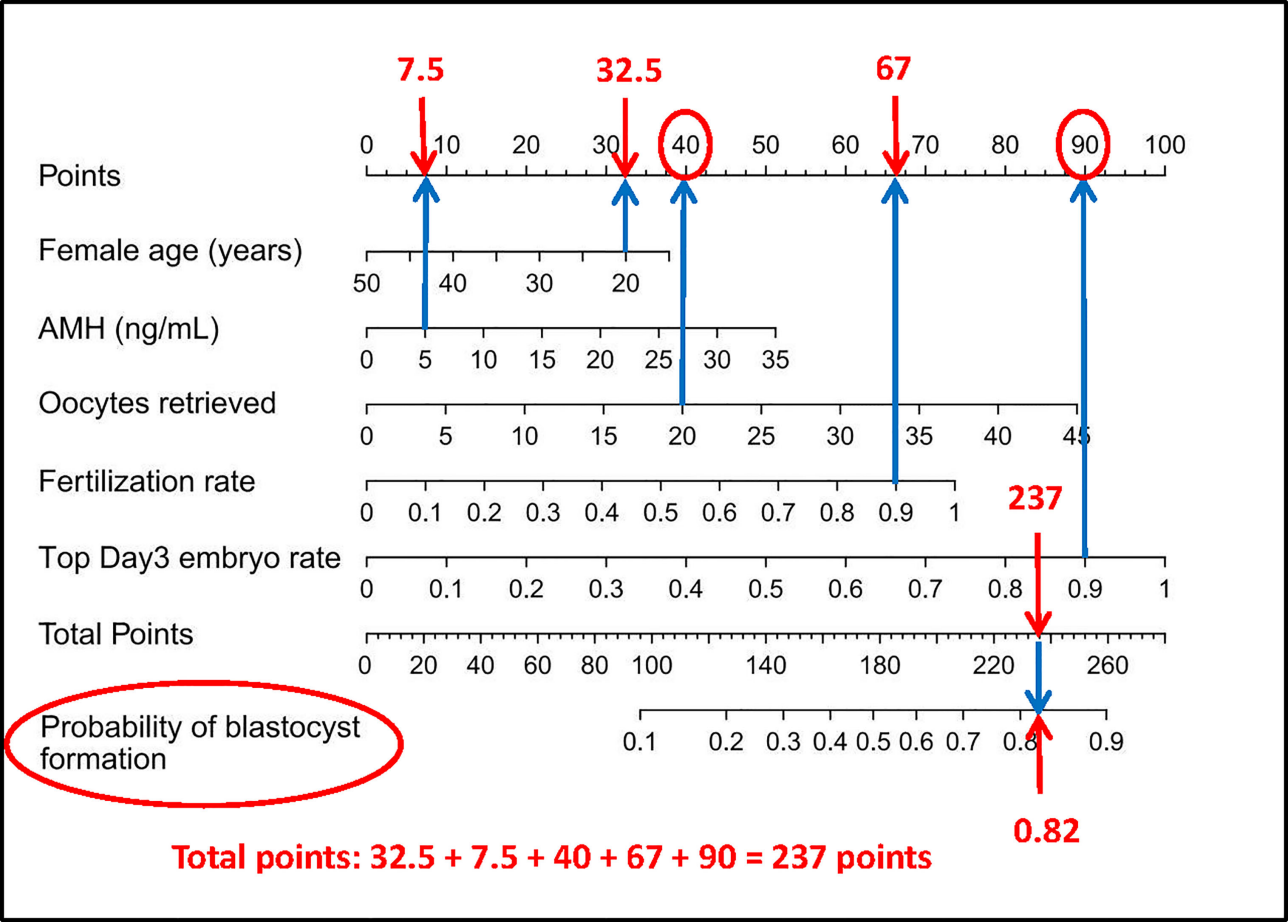
How to read the nomogram. For example, for a 20-year-old woman who suffers from tubal factor infertility (choose the nomogram of tubal factor group: A) with IVF treatment, AMH level of 5 ng/ml, 20 oocytes retrieved, 90% fertilization rate, and 90% top day 3 embryo rate, referring to the top scale, the scores of the above variables (blue arrow) are 32.5 points, 7.5 points, 40 points, 67 points, and 90 points, respectively (red arrow). Then refer to the total points to get the total score of 237 points, and the predicted probability of blastulation rate is 0.82. Finally, the clinician and the patient discuss whether blastocyst culture should be performed. AMH, anti-Mullerian hormone; IVF, *in vitro* fertilization.

Nomograms developed from the training sets in the three groups are shown in **Figure 3**. The AUC of the ROC curve for the tubal factor group model was 0.740 (95% CI: 0.724–0.757) in the training set and 0.735 (95% CI: 0.708–0.762) in the validation set. The AUC of the ROC curve for the PCOS group in the training set was 0.776 (95% CI: 0.749–0.803), while the AUC of the validation cohort was 0.787 (95% CI: 0.748–0.827). The AUC for the endometriosis group in the training set was 0.739 (95% CI: 0.686–0.792), and the AUC for the validation set was 0.750 (95% CI: 0.675–0.824). Collectively, the AUCs for the three groups indicated that the models exhibited fair performance (**Figure 4**). Moreover, calibration curves also showed good agreement between the observed and predicted probability for the tubal factor, PCOS, and endometriosis groups (**Figure 5**).Our nomograms were user-friendly; **Figure 6** shows graphical representations of the three models and how to use the nomograms.

## Discussion

Our analysis of infertile patients led to the generation of original models that can be used to predict the individual probability of extended culture to the blastocyst stage for IVF treatment in women with tubal factor infertility, PCOS, or endometriosis, depending on significant clinical and laboratory data. According to Dunnett’s t-test, the blastocyst formation rate in any two of the three groups was significantly different. Increased follicle recruitment and impaired oocyte maturation and development competence in PCOS women may be related to endocrine/paracrine factors with abnormal follicular secretion, metabolic dysfunction, and changes in the follicular microenvironment (19, 20). In a previous study, Heijnen et al. found that the fertility rate of PCOS patients was lower than of controls (21). Endometriotic tissues are known to produce reactive oxygen species (ROS), which have adverse effects on oocytes, sperm, embryonic microtubules, chromosomes, and DNA; furthermore, ROS can result in a decline in the quantity and quality of oocytes and embryos (22). These data suggest that it makes statistical and clinical sense to divide patients into three groups based on the etiology of infertility when analyzing blastocyst formation rate.

To increase the rate of blastocyst formation, the morphological assessment of cleavage stage embryos is a vital step of normal daily practice in the embryo laboratory (23). However, the most common and standard technique for embryo selection relies on highly subjective morphological assessments that are inaccurate. In recent years, various methods have emerged for embryo selection and have been widely adopted. It is possible to assess oocyte quality and embryo viability by measuring metabolites; this represents a non-invasive method and causes no additional damage to the embryo and oocyte (24). Time-lapse systems take digital images of embryonic stages at short time intervals so that an embryologist can assess the quality of the embryo without having to take it out of an incubator. However, these two methods not only require special instruments in the embryology laboratory, but they also cause unnecessary economic burden for the patients. In addition, several randomized controlled studies have been concluded that the widespread application of PGT incurs additional costs and invasiveness with limited benefits, especially for young women (25).

To reduce the costs of IVF treatment for infertile patients and reduce the risk of invasive injury to embryos, it is convenient and feasible to develop a predictive model based on clinical patient data to advise patients whether blastocyst culture is necessary. We constructed a predictive model using various significant risk factors related to blastocyst formation and then converted the prediction model into a nomogram. Nomograms were developed for the tubal factor, PCOS, and endometriosis training cohorts and then tested on the internal validation cohorts. Performance was evaluated using both calibration and discrimination (18). Clinicians can also obtain a total score for the outcome according to various basic patient variables included in the models such that patients can be told the probability of blastocyst formation.

A previous study predicted the cancellation rate of blastocyst transplantation by using a nomogram (12). However, this previous study did not consider either the etiology of female infertility separately or the influence of male factors on blastocyst formation; therefore, this previous model may have had a detrimental impact on the prediction of outcome. To improve the model, we analyzed the effect of female factors on blastocyst formation. Moreover, considering the influence of different causes of infertility on blastocyst formation, we divided patients into three groups according to the common causes of female infertility: a tubal factor group, a PCOS group, and an endometriosis group.

ICSI cycles were excluded from the inclusion criteria because the use of ICSI cycles may include cycles with poor sperm quality; ICSI can also have adverse effects on an embryo. A previous study concluded that ICSI led to a lower rate of blastocyst development in men undergoing ICSI for severe male factor infertility when comparing the quality and development of blastocysts derived from conventional oocyte insemination with those derived from ICSI (26).

The covariables included in our models were female age, serum AMH levels, the number of oocytes retrieved, the rate of fertilization, and the rate of top-quality day 3 embryos. The risk factors for our models are clinically significant and concordant with the published data. Female age has been reported to be an important prognostic factor for preimplantation embryo development (27). Maternal aging leads to impairments in chromosomal segregation, thus leading to an increased risk of aneuploidy and polyploidy. Moreover, cytoplasmic maturation is also age-related. Oocyte retrieval is known to be one of the most critical independent factors associated with blastulation. For example, Vermey et al. demonstrated that the number of oocytes retrieved was positively correlated with the number of top-quality embryos; similar associations were observed for MII oocytes, 2PN oocytes, and euploid embryos (28). In addition, not only the number of total or mature oocytes, but also the number of zygotes and cleaved embryos, has been shown to be associated with live birth rate in fresh donor oocyte IVF cycles (29); these findings are consistent with our present analysis. However, a previous study reported that if more than 15 oocytes were retrieved, then the risk of ovarian hyper-stimulation syndrome (OHSS) increased significantly; furthermore, the live birth rate did not increase in fresh autologous IVF cycles (30). Other research has shown that the incidence of severe OHSS correlates significantly with the number of oocytes retrieved, especially when 18 or more oocytes are retrieved (31). AMH, a member of the transforming growth factor-β superfamily of growth factors, is secreted by granulosa cells surrounding the pre-antral and early antral follicles and plays a key role in folliculogenesis. A previous study demonstrated that plasma levels of AMH did not change during the follicular phase of the menstrual cycle (32). Compared with women with elevated levels of FSH but lower levels of AMH, women with variable serum AMH levels that are greater than or equal to 0.6 ng/ml had more oocytes retrieved, more embryos on day 3, and a lower transfer cancellation rate (33). Another study concluded that serum levels of AMH were associated with specific oocyte dysmorphisms and an average oocyte quality index (34). The rates of fertilization and high-quality embryos are crucial aspects affecting blastocyst formation. The higher the fertilization rate and the rate of high-quality day 3 embryos, the higher the quality of the embryos and the higher the rate of blastocyst formation.

We did not incorporate female BMI into our model. Although pre-pregnancy excessive weight and obesity are associated with higher risks of preterm birth, macrosomia, and large for gestational age singletons conceived by ART (35), research has also shown that obesity does not have any significant effect on markers of oocyte quality or clinical pregnancy rates (36). Furthermore, female BMI is not related to oocyte dysmorphism (37). In a cohort study of 2,722 cycles, researchers concluded that an oocyte donor BMI up to 28 kg/m^2^ did not influence reproductive outcomes in recipients (38). A retrospective study of 6,500 IVF/ICSI cycles concluded that female obesity changed the uterine environment to impair IVF outcomes, although embryo quality was not affected (39). Collectively, these studies indicate that BMI has no significant effect on embryo quality. In the present study, we do not consider that the total dose of Gn has significant statistical difference on blastocyst formation. A previous study showed that the dose of Gn had no relationship with the rate of embryo aneuploidy and clinical pregnancy outcome in a PGT cycle (38); these findings concur with our present results.

**Figure 7:**
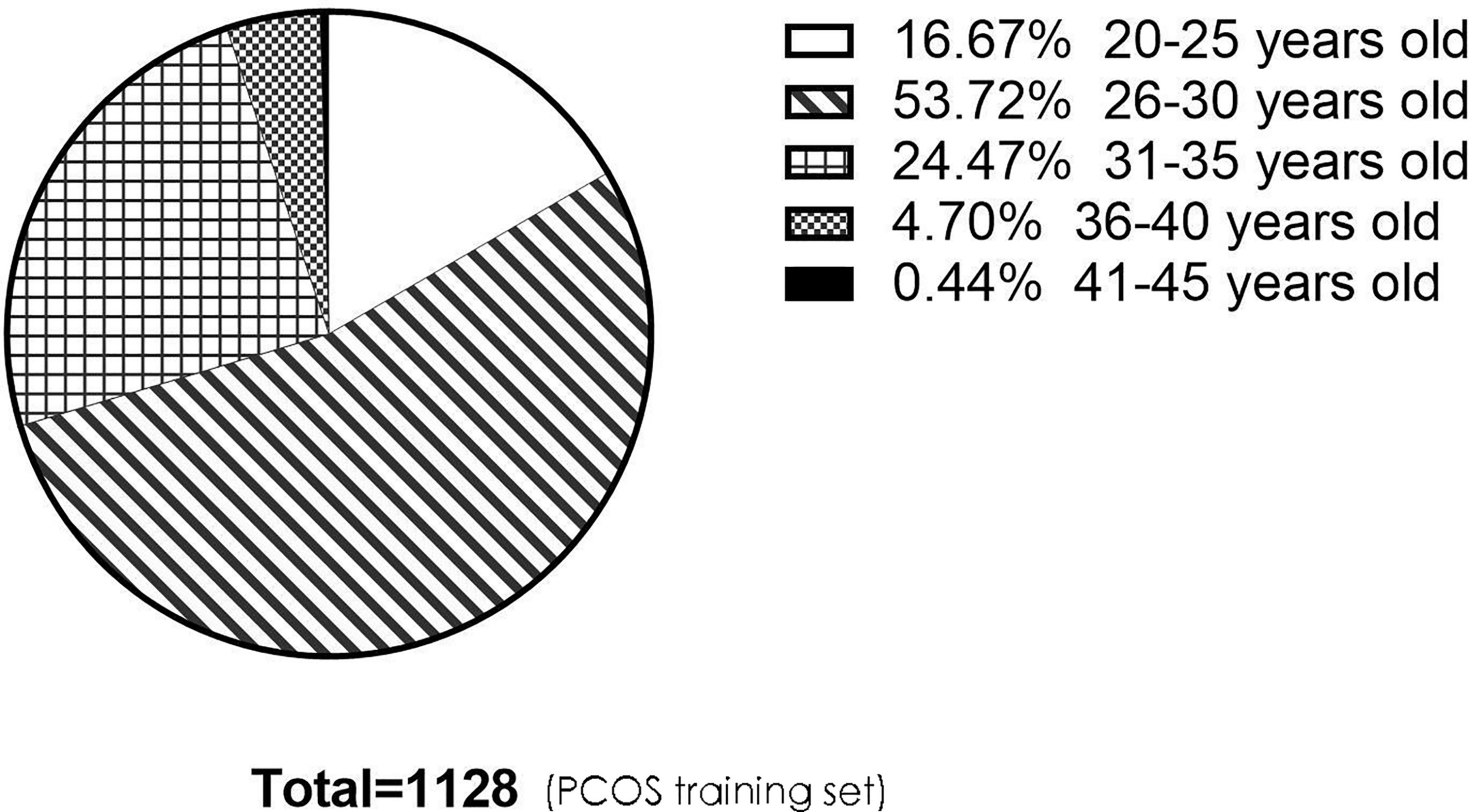
The pie chart describes the age distribution of patients in the PCOS training set. There were 188 (16.67%), 606 (53.72%), 276 (24.47%), 53 (4.70%), and 5 (0.44%) people aged 20–25, 26–30, 31–35, 36–40, and 41–45, respectively. There were 1,070 people under the age of 35, accounting for 94.86%. PCOS, polycystic ovary syndrome.

Although the age and serum AMH level in the PCOS and endometriosis groups were clinically relevant in terms of blastocyst formation, these two variables were not significantly associated with blastocyst formation when screening for variables using backward stepwise regression. There may be several reasons that could explain this finding. First, the mean age of patients in the PCOS training set was 28 years (**Table 2**), while the mean age of patients in the tubal factor training set was 31 years (**Table 1**), thus indicating that patients in the PCOS group received IVF treatment earlier than those in the tubal factor group. Second, **Figure 7** shows that 94.86% of patients in the PCOS group were younger than 35 years of age, thus weakening the negative effect of age on blastocyst formation in the construction of the predictive model, although female age was negatively correlated with oocyte quality. Lehmann et al. reported that serum AMH was a marker of oocyte quality and noted a reduction in implantation rate and pregnancy rate when levels of AMH were lower than 1 ng/ml (40). The mean level of serum AMH in patients in the PCOS training cohort was 8.46 ng/ml (**Table 2**); this is compared with 3.50 ng/ml in the tubal factor group (**Table 1**). Pigny et al. demonstrated that the serum AMH levels of PCOS patients were two- to threefold higher than those in a control group (41); these findings concurred with our present results. Therefore, the high levels of serum AMH in the PCOS group may weaken its adverse effects on oocyte quality, resulting in no statistically significant difference when the data of PCOS group were analyzed. However, in the endometriosis group, it is possible that the sample size was too small to yield a statistically significant difference for serum AMH level and age; these results may have been different if the sample size had been larger. Meanwhile, we compared the predictive power of the current model with that of the model excluding female age and serum AMH. According to **Table 5**, we inferred that the prediction ability of the current model was better than that of the model created without female age and serum AMH.

**Table 5:** Comparison of performance among prediction models in endometriosis group.

Variables	AUC (95% CI)	Hosmer–Lemeshow test (p-value)
Oocytes retrieved + Fertilization rate + Top day 3 embryo rate	0.738 (0.684–0.791)	0.101
Oocytes retrieved + Fertilization rate + Top day 3 embryo rate + Female age + AMH	0.739 (0.686–0.792)	0.195

Hosmer–Lemeshow test (p-value): p > 0.05 indicates good calibration degree. When the p-value is large, it indicates that the model fits well and the model has strong prediction ability.

AUC, area under the curve, AMH, anti-Mullerian hormone.

One of the main limitations of this study is that the data used to construct the predictive models were only collected from a single center and that the models were validated using independent internal data. However, we did not use external data from other reproductive centers to validate our models. Validating predictive models in an external cohort is necessary before they can be translated to the clinic; this is due to the lower performance of most predictive models when tested outside of the source population (42). Another major limitation of the study is mentioned in the methodology itself: embryos that were extended culture to the blastocyst stage were the remaining embryos that showed normal fertilization after fresh transfer and freezing on day 3, although they were randomly assigned to the training and validation sets in a 7:3 ratio. However, if the overall study design is to be more rigorous, the whole cohort of zygotes should be cultured to the blastocyst stage in each case group. Furthermore, the predictive models constructed in this study only predicted the average blastocyst formation rate for the entire embryo group, not for individual embryos and aneuploidy, and these applied only to specific female infertility factor patient groups. In addition, this was a retrospective study; thus, we cannot exclude the possibility of potential bias.

In conclusion, we developed three discrimination models to predict the probability of extended culture to the blastocyst stage for females undergoing IVF treatment with tubal factor infertility, PCOS, or endometriosis. Interestingly, this is the first study on blastocyst formation to take into account many female factors that may influence blastocyst formation and to exclude male factors and ICSI. Furthermore, the nomograms in this study were applicable to three common causes of female infertility. These models exhibited fair performance and were well calibrated. If these predictive models are confirmed by external validation and the sample size is increased in the PCOS and endometriosis groups, they will represent convenient and practical tools to help guide the management of patients with regard to extended culture to the blastocyst stage.

## Data Availability Statement

The original contributions presented in the study are included in the article/supplementary material. Further inquiries can be directed to the corresponding author.

## Ethics Statement

All the patients had been informed that their personal medical history information would be used and the study was approved by the Ethics Committee of the First Affiliated Hospital of Zhengzhou University (2021-KY-0023-001).

## Author Contributions

HJ and XS conceived the study, analyzed the data, and wrote the paper. WS and YL collected and analyzed the data. LQ and FZ validated the data and modified the manuscript. All authors contributed to the article and approved the submitted version.

## Funding

This work was supported by grants from the Natural Science Foundation of Henan Province of China (General Program) (No. 202300410467) and Key Projects of Medical Science and Technology in Henan Province of China (No. SBGJ2020002053).

## Conflict of Interest

The authors declare that the research was conducted in the absence of any commercial or financial relationships that could be construed as a potential conflict of interest.

## Publisher’s Note

All claims expressed in this article are solely those of the authors and do not necessarily represent those of their affiliated organizations, or those of the publisher, the editors and the reviewers. Any product that may be evaluated in this article, or claim that may be made by its manufacturer, is not guaranteed or endorsed by the publisher.
